# Demographic Characteristics and Treatment Outcomes of Advanced Renal Cell Carcinoma With Clear Cell Histology: A Single-Center Experience From India

**DOI:** 10.7759/cureus.61978

**Published:** 2024-06-08

**Authors:** Somnath Roy, Bivas Biswas, Deepak Dabkara, Sandip Ganguly, Joydeep Ghosh, Arnab Bhattacharjee, Kuntal Ray, Sayan Mandal, Yesha S Patel, Souhita Pal, Jagriti Karmakar, Anindita Mitra, Rupsa Bakshi, Sumit Mukhopadhyay, Sujoy Gupta

**Affiliations:** 1 Medical Oncology, Tata Medical Center, Kolkata, IND; 2 Radiodiagnosis, Tata Medical Center, Kolkata, IND; 3 Urology, Tata Medical Center, Kolkata, IND

**Keywords:** outcome, response, pazopanib, clear cell, renal cell carcinoma

## Abstract

Background

Treatment of metastatic renal cell cancer (mRCC) has revolutionized with the introduction of anti-VEGF tyrosine kinase inhibitors (TKIs) and immune checkpoint inhibitors (ICIs). There is limited data in the literature on the outcomes of Indian patients treated with TKI. Here, we report the outcome of mRCC treated with first-line TKI in a resource-poor setting.

Material and methods

This is a single-center retrospective study of clear cell mRCC treated with first-line TKI from June 2012 to December 2022. Demographic characteristics and treatment details, including outcome data, were captured from electronic medical records. Patients who received at least one week of therapy were eligible for survival analysis.

Results

A total of 345 patients with metastatic clear cell histology were analyzed, with a median age of 61 years (range: 20-84 years). One hundred and eighty patients (52%) underwent nephrectomy before systemic therapy. The majority received pazopanib (257 patients, 75%), followed by sunitinib (36 patients, 10%) and cabozantinib (21 patients, 6%); 145 (45%) patients required dose interruption, and 143 (43%) required dose modification of TKI for adverse events. After a median follow-up of 44 months, the median progression-free survival (PFS) was 20.3 months (95% CI: 17.8-24.8), and the median overall survival (OS) was 22.7 months (95% CI: 18.8-28.3). In the poor-risk International Metastatic Renal Cell Carcinoma Database Consortium (IMDC) group, no prior nephrectomy emerged as an independent poor-risk factor for both PFS and OS in multivariate analysis.

Conclusion

This is the largest single-center cohort of clear cell mRCC from Asia. Median PFS was 20.3 months with predominantly TKI monotherapy. In the poor-risk IMDC group, no prior nephrectomy emerged as an independent poor-risk factor for both PFS and OS.

## Introduction

Clear cell histology is the most common variety of kidney cancer, with a historical approximate five-year survival rate of 3% with interferon-alpha [1]. The outcome has substantially improved with the development and introduction of novel targeted therapies for metastatic renal cell carcinoma (mRCC) with clear cell histology. The most important prognostic factor to predict outcome in newly diagnosed mRCC is baseline risk stratification [2]. The International Metastatic Renal Cell Carcinoma Database Consortium (IMDC) is the most commonly used validated risk scoring system to predict outcomes and customize treatment in newly diagnosed mRCC [2,3].

For nearly two decades, the mainstay of treatment for mRCC was a tyrosine kinase inhibitor (TKI) of vascular endothelial growth factor (VEGF). Two landmark phase 3 clinical trials established the role of two anti-VEGF TKIs (sunitinib and pazopanib) in the management of mRCC [4-6]. Later, cabozantinib, another multikinase inhibitor (including VEGF), showed superior progression-free survival (PFS) and overall response (OS) rates compared to sunitinib in a phase 2 clinical trial in intermediate and poor-risk clear cell mRCC [7]. Subsequently, immune checkpoint inhibitors (ICIs) showed excellent results in the second-line setting and later moved to first-line treatment with the combination of VEGF TKI, which had a better response rate and durable survival outcome [8,9].

Indian patients were not included in any of those landmark registration trials. There is a huge unmet need in the treatment of mRCC patients in India, as access to these novel targeted therapies is limited due to various reasons. There is a paucity of data regarding demography and treatment outcomes in Indian patients with mRCC [10,11]. Here, we report real-world data on the demographic profiles, clinical characteristics, and treatment outcomes of patients with clear cell mRCC treated with anti-VEGF TKI at our center.

## Materials and methods

Primary objectives

The study focuses on the demographic characteristics and treatment outcomes with first-line TKI in mRCC.

Secondary objectives

We measured survival in terms of PFS, OS, and risk stratification-based outcomes.

Study details

This is a single-center retrospective, non-interventional, observational study of mRCC with clear cell histology treated with first-line TKI from June 2012 to December 2022 at Tata Medical Center, Kolkata, India. Consecutive patients meeting the eligibility criteria were enrolled and followed up until March 2023. 

Eligibility criteria

All patients aged ≥18 years with a histopathological diagnosis of advanced or mRCC (stage IV as per the American Joint Committee of Cancer Staging Sixth and Seventh Edition [12]) registered in the Department of Medical Oncology were considered eligible for this study. Patients with measurable diseases as per Response Evaluation Criteria in Solid Tumors (RECIST v1.1) [13] were included in this study. Patients with a non-clear histology were excluded. Patients with inadequate medical records and those who did not take any treatment after the initial evaluation were excluded from the survival analysis. Because of the retrospective nature of the study, a waiver of consent was received from the Institutional Review Board as per institutional policy (Institutional Ethics Committee (IEC) protocol waiver no. EC/WV/TMC/09/23).

Treatment and response evaluation

In the initial few years, patients were treated with first-line VEGF TKI (either sunitinib or pazopanib) as monotherapy for the favorable and intermediate risk groups and everolimus for the poor risk group. Subsequently, cabozantinib was introduced for intermediate- and poor-risk groups in mRCC (after it was launched in India). A few affordable patients with intermediate- and poor-risk mRCC were treated with first-line TKI and ICI combinations. Response evaluation was done with appropriate imaging techniques at least three months after starting treatment or earlier if indicated. Thereafter, period imaging was done every three to six months, or as clinically indicated. Response evaluation was done using both RECIST (version 1.1) and Morphology, Attenuation, Size, and Structure (MASS) criteria. The clinical benefit rate was represented by the response to therapy (complete and partial response plus stable diseases). Patients were treated until there was documented disease progression or unacceptable toxicity. Dose reductions and dose interruptions were done according to standard guidelines as per the severity of adverse effects.

Data collection and data source

Demographic features, treatment details, baseline IMDC and Memorial Sloan Kettering Cancer Center (MSKCC) risk grouping, nephrectomy, dose modifications of TKI, treatment response, pattern of progression, and outcome data were collected for analysis. All data were captured from the electronic medical records of the hospital.

Statistical methods

There was no formal study sample size, as it was a single institutional, non-interventional, single-arm retrospective chart review. Descriptive statistics, tables, and charts were used to analyze the demographic, clinical, and treatment-related variables. Kaplan-Meier survival curves were used for survival estimates. Factors like the IMDC risk group, MSKCC risk score, and nephrectomy status were analyzed for predicting PFS and OS by univariate Cox regression analysis. Factors with a p-value of ≤0.1 in univariate analysis were taken into stepwise multivariate analysis. Patients who received at least one week of TKI therapy were taken for survival analysis. Data were censored on March 31, 2023. Patients who were lost to follow-up were censored at the date of last contact or follow-up. The PFS was calculated from the date of initiation of systemic treatment to the date of disease progression or death from any cause. The OS was calculated from the date of diagnosis to the date of death from any cause. Patients who were lost to follow-up or who had abandoned treatment were also included in the PFS and OS analyses, and the outcomes for these patients were determined by telephone contact. Stata/SE 13.0 (StataCorp, College Station, Texas) was used for statistical analysis.

## Results

Demographic details

**Figure 1:**
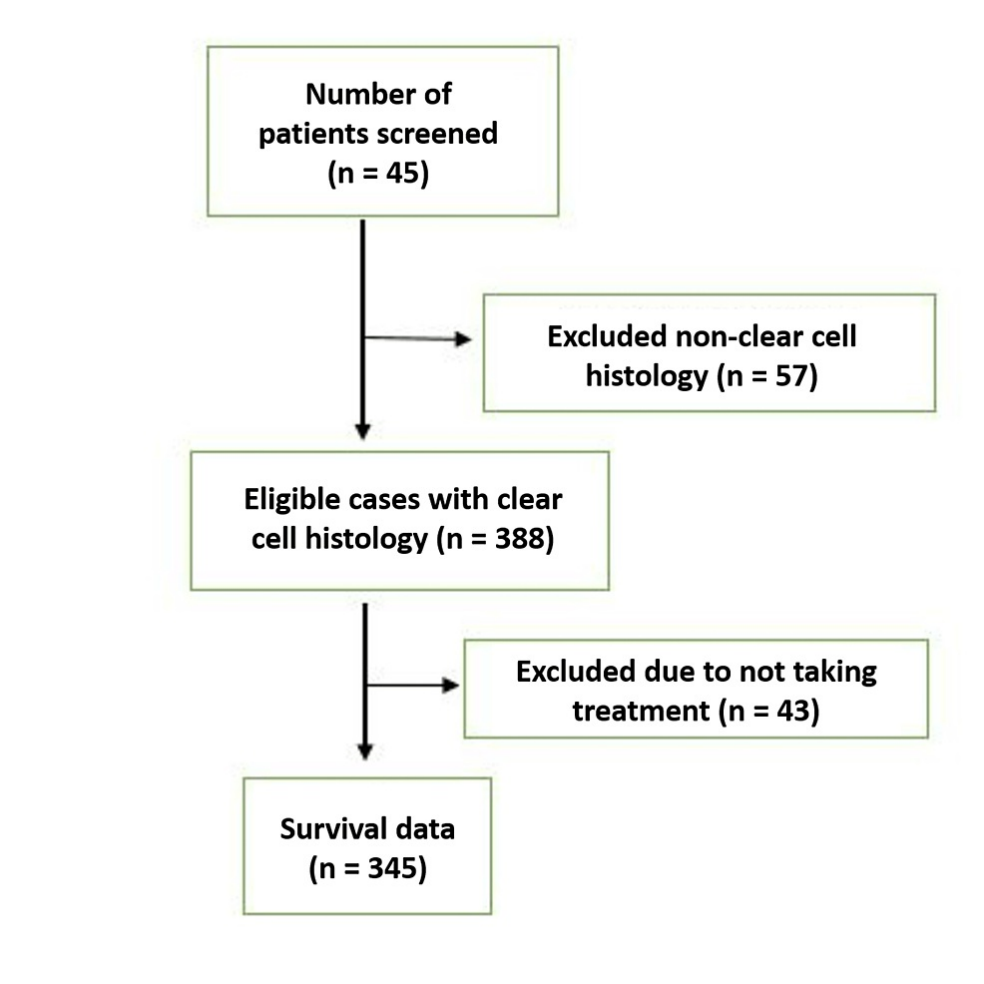
A flowchart showing patient selection, exclusion, and enrollment in the study

During the study period, a total of 445 cases of mRCC were initially screened, and out of them, 57 were excluded due to non-clear cell histology. Out of 388 eligible cases of clear cell mRCC, 43 patients didn't take any treatment and were lost to follow-up. Survival analysis was done for 345 patients (Figure 1). The median age was 61 years (range: 20-84 years), with a male-female ratio of 279:66. Only 10% presented with a classical triad of symptoms (fever, abdominal pain, and hematuria), and the most common sites of metastasis were the lung (247 patients, 72%), bone (123 patients, 36%), and distant lymph node (64 patients, 19%). Demographic details are summarized in Table 1, and Figure 1 shows the flowchart of patient enrollment.

**Table 1:** Demographic profiles of the study group IDMC: International Metastatic RCC Database Consortium; MSKCC: Memorial Sloan Kettering Cancer Center

Variables	N = 345 (%)
Age (in years, median; range)	61 years; 20-84
Gender	
Male	279 (81)
Female	66 (19)
Smoking status	
Smoker	166 (48)
Non-smoker	81 (24)
Unknown	98 (28)
Triad of symptoms	
Yes	34 (10)
No	311 (90)
Site of metastasis	
Lung	247 (72)
Liver	61 (18)
Bone	123 (36)
Brain	18 (5)
Distant lymph nodes	64 (19)
IMDC favorable risk	18 (5)
Intermediate risk	159 (46)
Poor risk	131 (38)
Unknown	37 (11)
MKSCC	
Favorable risk	4 (1)
Intermediate risk	158 (46)
Poor risk	105 (30)
Unknown	78 (23)
Nephrectomy	
Yes	180 (52)
No	165 (48)

Risk stratification

Favorable, intermediate, and poor risk were 5%, 46%, and 38%, respectively, as per IMDC criteria, and 1%, 46%, and 30%, respectively, as per MSKCC criteria. The IMDC and MSKCC data were missing for 11% and 23% of patients, respectively (Table 1).

Treatment and response

Among the participants, 180 (52%) patients underwent nephrectomy (either cytoreductive nephrectomy for localized disease or removal of a kidney tumor with palliative intent in a metastatic setting) before the start of systemic therapy. The majority of patients received pazopanib (n = 257; 75%), followed by sunitinib (n = 36; 10%), cabozantinib (n = 21; 6%), and others (n = 18; 5%). Only 13 (4%) patients received TKI plus ICI combinations (Table 2).

**Table 2:** Treatment details TKI: tyrosine kinase inhibitor; ICI: immune checkpoint inhibitor

Variables	N= 345 (%)
First-line treatment	
Pazopanib	257 (75)
Sunitinib	36 (10)
Cabozantinib	21 (6)
TKI + ICI combination	13 (4)
Others	18 (5)
Best response	
Complete response	3 (1)
Partial response	113 (33)
Stable diseases	161 (47)
Progressive diseases	51 (14)
Unknown	17 (5)
Dose reduction (n=330)	
Yes	143 (43)
No	187 (57)
Dose interruption (n=323)	
Yes	145 (45)
No	178 (55)
Subsequent line of therapy	
Everolimus	59 (17)
Lenvatinib + everolimus	33 (9)
Nivolumab	6 (2)
Pazopanib	4 (0.57)
Axitinib	7 (2)
Erlotinib + bevacizumab	1 (0.28)
Cabozantinib	18 (5)
TKI + ICI	7 (2)

Tyrosine kinase inhibitor dose intensity and modification

Patients received different starting doses of TKI depending on their Eastern Cooperative Oncology Group Performance Status (ECOG PS), age, comorbidities, and organ functions. The majority of patients received standard recommended doses of different TKIs (Table 3). Of the patients, 145 (45%) underwent dose interruption due to adverse effects (AE), and 143 (43% of patients) required dose reductions or modifications due to AEs. Details of second-line therapy after progression are documented in Table 2. Everolimus was the most common (44%) second-line treatment, followed by lenvatinib plus everolimus (24%).

**Table 3:** Dose modifications and interruptions

Tyrosine kinase inhibitors	Starting dose daily (n)		
Pazopanib (n=257)	800 mg (n=208)	600 mg (n=38)	400 mg (n=11)
First dose reduction	600mg (n= 47)	400mg (n= 18)	200 mg (n=1)
Second dose reduction	400mg (n= 37 )	--	--
Third dose reduction	200mg (n=12)	--	--
Sunitinib (n=36)	50 mg (n=29)	37.5 mg (n=4)	25 mg (n=3)
First dose reduction	37.5 mg (n=7 )	25mg (n= 1)	--
Second dose reduction	25 mg (n=8 )	--	--
Cabozantinib (n=21)	40 mg (n=20)	20 mg (n=1)	--
First dose reduction	20 mg (n= 6)	--	--

Treatment outcomes and survival analysis

**Figure 2:**
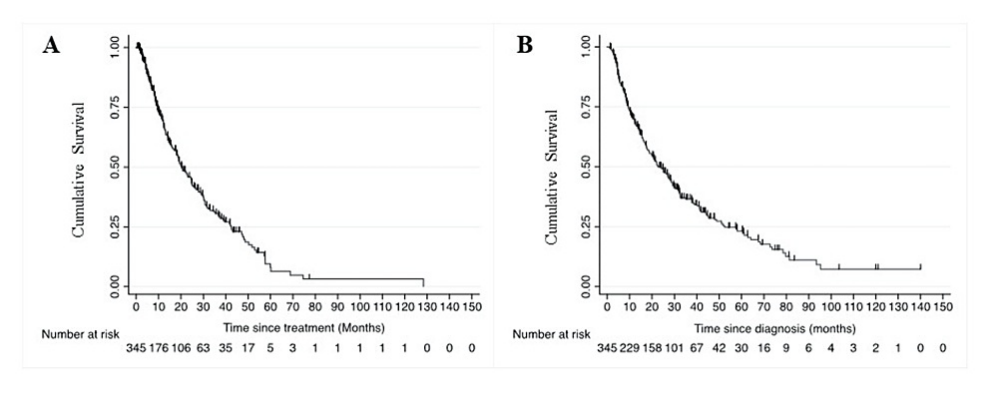
Kaplan-Meier graph showing the median PFS of the whole cohort (panel A) and the median OS of the whole cohort (panel B). PFS: progression-free survival; OS: overall survival

**Figure 3:**
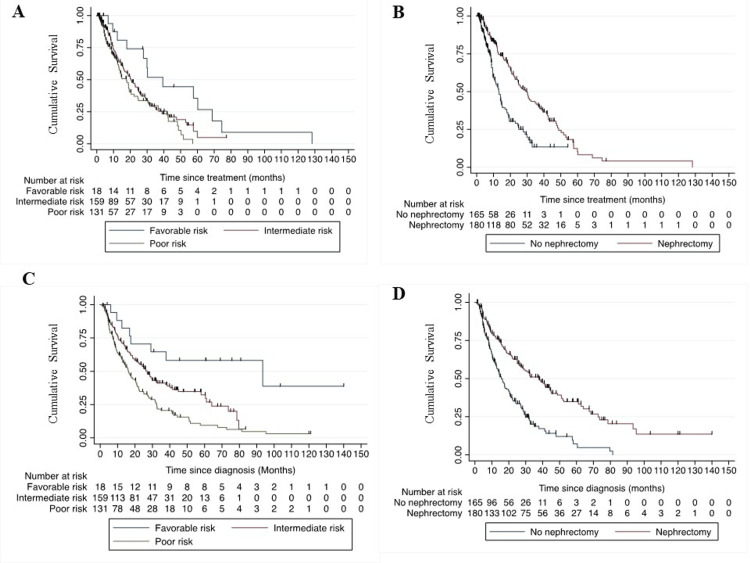
Kaplan-Meier graph showing median PFS as per the IMDC risk group (panel A), prior nephrectomy (panel B), and median OS as per the IMDC risk group (panel C) and prior nephrectomy (panel D). PFS: progression-free survival; IMDC: International Metastatic Renal Cell Carcinoma Database Consortium; OS: overall survival

The overall response rate to first-line treatment was 34%, and the clinical benefit rate was 81% (Table 2). After a median follow-up of 44 months, the median PFS was 20.3 months (95% CI: 17.8-24.8) (Figure 2A), and the median OS was 22.7 months (95% CI: 18.8-28.3) (Figure 2B). Univariate analysis showed an estimate of inferior PFS for patients without nephrectomy (p<0.001), IMDC poor-risk (p=0.003), and intermediate-risk group (p=0.022), and inferior OS for patients without nephrectomy (p<0.001), IMDC poor-risk (p=0.001), and intermediate-risk group (p=0.02) (Tables 4-5). The multivariate logistic regression analysis identified the poor-risk IMDC group and no prior nephrectomy as independent poor-risk factors for PFS and OS (Tables 4-5, Figures 3A-3D).

**Table 4:** Univariate and multivariate analysis for progression-free survival (PFS) *The p-value was derived from the Cox regression analysis. CI: confidence interval; HR: hazard ratio; IDMC: International Metastatic RCC Database Consortium; MSKCC: Memorial Sloan Kettering Cancer Center

Variable	HR(95%CI)	Median PFS in months (95% CI)	p-value*	HR (95% CI)	p-value*
Univariate analysis				Multivariate analysis	
IMDC risk group	--	--	--	--	--
Favorable (n=18)	1	39.4 (17.9 - 68.8)	--	1	--
Intermediate (n=159)	2.1 (1.1 - 4)	20.3 (15.8 - 25)	0.022	1.9 (1 - 3.6)	0.058
Poor (n=131)	2.7 (1.4 - 5.3)	17.6 (13- 20.4)	0.003	2.4 (1.2 - 4.7)	0.010
MSKCC risk group	--	--	--	--	
Favorable (n=4)	1	16.1 (16.1- NR)	--		
Intermediate (n=158)	1.5 (0.4 - 6.2)	24.4 (18.9 - 29.8)	0.56		
Poor (n=105)	2.7 (0.7 - 11.1)	12.4 (9.6 - 17.6)	0.17		
Nephrectomy	--	--	--	--	--
Not done (n=165)	1	13.2 (10.2 - 14.9)	--	1	--
Done (n=180)	0.5 (0.3 - 0.6)	29.9 (22.8 - 36.7)	<0.001	0.48 (0.4 - 0.7)	<0.001

**Table 5:** Univariate and multivariate analysis for overall survival (OS) *The p-value was derived from the Cox regression analysis. CI: confidence interval; HR: hazard ratio; IDMC: International Metastatic RCC Database Consortium; MSKCC: Memorial Sloan Kettering Cancer Center

	Univariate analysis			Multivariate analysis	
Variable	HR(95%CI)	Median OS in months (95% CI)	p-value*	HR (95% CI)	p-value*
IMDC risk group	--	--	--	--	--
Favorable (n=18)	1	93.4 (16. 9 - NR)	--	1	--
Intermediate (n=159)	2.4 (1.1 - 4.5)	26.9 (19.8 - 32.4)	0.020	2.1 (1 - 4.3)	0.052
Poor (n=131)	3.9 (1.9 - 8)	16.3 (12.8 - 21)	0.001	3.2 (1.5 - 6.6)	0.002
MSKCC risk group	--	--		--	
Favorable (n=4)	1	21.6 (12-5 - NR)			
Intermediate (n=158)	0.9 (0.3 - 2.8)	29.7 (24.2 - 40.2)	0.84		
Poor (n=105)	1.7 (0.5 - 5.4)	16.3 (12.8 - 19.7)	0.37		
Nephrectomy	--	--	--	--	--
Not done (n=165)	1	15.7 (12.8- 20)	--	1	--
Done (n=180)	0.4 (0.3 - 0.6)	37.7 (27- 44.9)	<0.001	0.5 (0.4 - 0.7)	<0.001

## Discussion

To the best of our knowledge, this is the largest single-center Indian dataset analyzing the demographic profiles, treatment responses, and survival outcomes of patients with clear cell mRCC. The majority (91%) of the patients received anti-VEGF TKI with an estimated median PFS of 20.3 months in a non-trial, real-world setting within the Indian population, and that too in a cohort with only 5% of patients in the IMDC-favorable risk group.

There was a paradigm shift over recent years in the management of mRCC after the success story of ICIs and combination approaches with anti-VEGF TKI. Evidence from randomized control trials (RCTs) showed that the estimated average median PFS was near about 12 months with first-line TKI monotherapy (either sunitinib or pazopanib) in mRCC, with a median OS around 28 months [4,5]. However, in developing countries like India, it is difficult to implement ICIs in routine practice due to the high cost of medicine, the national insurance plan, very poor coverage of personal health insurance, and poor regulation of government agencies regulating private insurance [14,15].

The demographic profile of our study was closer to the database of real-world studies from India and most RCTs [9-11]. The age of presentation, histology, site and number of metastases, and IMDC and MSKCC risk groups match the available published literature (Table 6). Generic sunitinib or pazopanib were not available during the period from 2012 to 2019. The cost of the innovator molecules (357 USD for pazopanib and 1,700 USD for sunitinib) was a major challenge for most of our patients [10]. Subsequently, from 2020 onwards, generic pazopanib and sunitinib arrived, and later, generic cabozantinib was launched in India in 2021. For that reason, most of our patients (75%) were treated with pazopanib, which was less expensive than others. Due to financial constraints and a limited access program, it was a major challenge to offer TKI plus ICI or double ICI in combination to our patients. Despite this limitation, the clinical benefit rate (81%) in our study was quite impressive and even higher than published data from Asia Pacific countries [10, 16-18].

**Table 6:** Real-world evidence of advanced clear cell carcinoma treated with first-line TKI *In combination with immune checkpoint inhibitor IDMC: International Metastatic RCC Database Consortium; MSKCC: Memorial Sloan-Kettering Cancer Center; OS: overall survival; PFS: progression-free survival; TKI: tyrosine kinase inhibitor

No.	Name of the author	No. of patients	Histology	Risk score	TKI used	Median PFS month	Median OS month
1	Erman et al., 2021 [16]	190	Clear cell (81%)	IMDC risk group	Pazopanib	10	Not reported
				Favorable: 8%			
				Intermediate: 42%			
				Poor: 11%			
				Unknown: 38%			
2	Schmidinger M et al., 2019 [19]	657	Clear cell or/predominate clear cell	IMDC risk group	Pazopanib	10.3	29.9
				Favorable: 5%			
				Intermediate: 52%			
				Poor: 23%			
				Unknown: 20%			
3	Staehler M et al., 2021 [20]	60	Clear cell	MSKCC risk	Pazopanib	4.5	9.3
				Intermediate: 25%			
				Poor: 67%			
				Unknown: 7%			
4	Schmidinger M et al., 2020 [21]	467	Not reported	IMDC risk group	Sunitinib	10.4	34
				Favorable: 15%			
				Intermediate: 25%			
				Poor: 10%			
5	Savard MF et al., 2020 [22]	1769	Clear cell	IMDC risk group	Sunitinib	8.1	28.6
				Favorable: 18%			
				Intermediate: 58%			
				Poor: 24%			
6	Krishna VM et at., 2013 [17]	59	Clear cell	Not reported	Sunitinib	11.4	22.6
7.	Rudresha AH et al., 2017 [18]	40	Clear cell	MSKCC group	Sunitinib and pazopanib	10.8	19.1
				Favorable: 7.5%			
				Intermediate: 62%			
				Poor: 30%			
8.	Ramaswamy A et al., 2017 [10]	212	Clear cell (68%)	IMDC risk group	Sunitinib (40%)	7.11	12.9
				Favorable: 19%	Pazopanib (3%)		
				Intermediate: 44%	Sorafenib (38%)		
				Poor: 28%			
9.	Present study	345	Clear cell	IMDC risk group	Sunitinib (10%)	20.3	22.7
				Favorable: 5%	Pazopanib (75%)		
				Intermediate: 46%	Cabozantinib (6%)		
				Poor: 38%	Other TKI* (4%)		
				MSKCC group			
				Favorable : 1%			
				Intermediate: 46%			
				Poor: 30%			

The MSKCC risk model was developed from data collected from clinical trials during the era of interferon alfa. Later, as treatment was revolutionized by anti-VEGF TKI, the more contemporary IMDC model was used and replaced the MSKCC model for deciding first- and second-line therapy [3]. However, there was a limitation on the risk stratification of each patient, like in India, where iron deficiency anemia is very common. In our analysis, IMDC risk scoring emerged as a better prognostic tool than MSKCC scoring (Tables 4-5).

The role of nephrectomy in the era of ICIs is not clearly defined because most of the ICI trials included patients with prior nephrectomy. Studies showed that patients with IMDC intermediate-risk/poor-risk on anti-VEGF TKI do not benefit from nephrectomy [23,24]. Besides this, there is a lack of validated prognostic and predictive biomarkers to identify patients who might benefit from ICIs [25]. But in this study, significantly better median PFS (29.9 months) and median OS (37.7 months) were observed in those who underwent prior nephrectomy (Figure 3).

More than 80% of our patients started with standard daily doses of pazopanib and sunitinib similar to those seen in the pivotal studies, and cabozantinib started with a reduction of one dose level (40 mg/daily) to avoid toxicity. Approximately 45% of populations underwent subsequent dose reductions and interruptions due to AEs that are similar to those reported in the PARACHUTE and PRINCIPAL studies [16,19].

This was the first reported study from India where the demographic and treatment details of 345 patients with clear cell mRCC were analyzed. In addition, we tried to offer maximum treatment benefit in a financially challenged patient population, especially considering the cost of ICIs and innovator TKI molecules, with a reasonable response rate and survival outcome as compared with large-scale prospective evidence. When we stratified median PFS according to the IMDC risk model, we found that median PFS was 39.4 months in the favorable risk group, followed by 20.3 months for the intermediate and 17.6 months for the poor risk group, which was comparable with non-trial eligible patients from the IMDC database and real-world studies also [20-22, 26]. Again, the median OS was 22.7 months, which was nearly similar to patients from the real-world Renal Cell Carcinoma Outcomes Research Dataset (RECCORD) registry (median OS: 23.9 months) [27]. Only 135 (39%) patients received second-line and subsequent lines of therapy, as no suitable treatment was available after first-line failure. These factors can explain the very short gap between the median PFS and median OS in our cohort.

We acknowledge that it was a retrospective, non-interventional study. The other major limitations were the exclusion of non-clear cell histology due to heterogeneity of treatment, not being able to analyze the differential toxicity profiles of each TKI, the median duration of exposure to each dose level, the correlation of survival outcomes at each dose level, and cancer-specific mortality. With rapidly changing standards of care, most of our patients didn't receive ICI-based therapy either upfront or in subsequent lines of treatment.

## Conclusions

This study establishes a benchmark for a median PFS of 20.3 months for patients with clear cell mRCC treated with TKI monotherapy with a durable response rate as first-line therapy in a real-world setting from India in the context of a modern treatment landscape. Although ICI plus TKI combinations are the current standard of care in mRCC with clear cell histology, irrespective of the IMDC risk group, this study also demonstrated that TKI monotherapy with or without nephrectomy is an alternative treatment option. In the IMDC poor-risk group, no prior nephrectomy was identified as an independent poor-risk factor for both PFS and OS. However, there is an urgent need to establish a nationwide registry and implement treatment guidelines with the use of ICI and TKI combinations in resource-constrained settings for better outcomes.
